# Burdens of idiopathic developmental intellectual disability attributable to lead exposure from 1990 to 2021 and projection to 2035 in China: findings from the 2021 global burden of disease study

**DOI:** 10.3389/fpubh.2025.1562794

**Published:** 2025-05-09

**Authors:** Chaoqun Song, Feidan Deng, Lichun Qiao, Minghao Lin, Hui Zhang, Miaoqian Li, Changwei Zhao

**Affiliations:** ^1^Changchun University of Chinese Medicine, Changchun, China; ^2^School of Public Health, Xi’an Jiaotong University Health Science Center, Xi’an, China; ^3^The First Affiliated Hospital of Changchun University of Chinese Medicine, Changchun, China

**Keywords:** idiopathic developmental intellectual disability, Pb exposure, disability-adjusted life years, GBD2021, age-standardized DALY rate

## Abstract

**Objective:**

To analyze the burden and trend of idiopathic developmental intellectual disability (IDII) attributed to lead (Pb) exposure in China from 1990 to 2021 and to predict the trend from 2022 to 2035.

**Method:**

We used the Global Burden of Disease Study (GBD) 2021 data to estimate disability-adjusted life years (DALYs) and age-standardized DALY rate (ASDR) of IDII attributable to Pb exposure. The annual average percentage change (AAPCs) was estimated to evaluate the changing trend of IDII ASDR attributable to Pb exposure from 1990 to 2021. The age-period-cohort (APC) and Bayesian age-period-cohort (BAPC) was used to assess and predict changes in the DALYs of IDII attributable to Pb exposure.

**Result:**

From 1990 to 2021, the number of DALYs and rate of the total population, males and females in China showed a fluctuating and decreasing trend. The APC showed that the age deviation had an upward trend and then decreased, the period deviation showed an ‘*N*’ shaped trend, and the cohort deviation model showed a trend of first increasing and then decreasing. The BAPC model predicts that the number of DALYs and ASDR will continue to decline, with males declining faster than females, and that by 2035, females will have higher DALYs and ASDR than males.

**Conclusion:**

From 1990 to 2035, the burden of IDII attributed to Pb exposure in China showed a downward trend. But the DALYs and ASDR in females will be higher than that in males in 2035. It is essential to prioritize intervention, prevention, and control measures for females.

## 1 Introduction

Intellectual disability (ID) is a neurodevelopmental disorder with significant limitations in intellectual functioning and adaptive behavior, present in 1–3% of the general population (1). Unexplained ID was called idiopathic developmental intellectual disability (IDII) and accounts for about 50% of all cases (2, 3). Evidence suggests that the prevalence of mental disorders among individuals with intellectual disabilities is substantially higher compared with the general population (4). Inborn metabolic disorders, genetic mutations, and environmental and familial factors are recognized as contributors to the high prevalence of IDII (5, 6). A 35-year cohort study demonstrated that individuals with severe ID have significantly elevated mortality rates compared to the general population, with few surviving to advanced age (7). A study of Chinese children found that low levels of lead (Pb) exposure were associated with innate cognitive abilities underlying language development (8). These findings underscore the need for governments and international health organizations to prioritize this vulnerable population through increased funding for preventive interventions and implementation of evidence-based treatment approaches.

Lead exposure is a severe environmental contaminant, and Pb can enter the bloodstream through respiratory exposure or oral ingestion, while blood Pb is excreted through urine and bile (9). Extensive epidemiological evidence has demonstrated that the most prominent clinical consequence of Pb exposure is neurotoxicity (10). The brain is the organ most sensitive to Pb toxicity, with chronic exposure associated with a variety of neuropsychiatric disorders, such as IDII, Parkinson’s disease, and attention deficit hyperactivity disorder (11, 12). Global epidemiological data estimate that approximately 600,000 new cases of children with ID attributable to Pb exposure occur each year, mainly in developing countries (13). Notably, retrospective cohort studies have demonstrated that Pb exposure is associated with an increased risk of psychiatric disorders in adults (14). Recent evidence reveals a declining trend in the incidence of Pb-associated intellectual disability in high-income countries, whereas low- and middle-income countries, among children and adolescents, still experience a substantial disease burden (15). A meta-analysis found that the Pb-exposed individuals had higher levels of Pb in their blood and lower overall intelligence quotient (IQ) scores than the control group (16).

A GBD study from 1991 to 2019 showed that China was the country with the highest number of deaths attributed to Pb exposure (12). However, although neurological impairment represents one of the primary health outcomes of Pb exposure, no study has systematically evaluated the burden of IDII attributable to Pb exposure in China or developed long-term projections. So, this study aims to examine the independent effects of age, period, and cohort Pb exposure in China between 1990 and 2021, using GBD 2021 data to compare these effects by age group and sex, and then projecting DALYs through 2035. The results of this study will provide a reliable epidemiological basis for further prevention of the impact of Pb exposure on IDII and promote the rational allocation of medical resources.

## 2 Materials and methods

### 2.1 Sources

GBD2021 database (GHDx^1^) was used to extract data on MD attributable to Pb exposure from 1990 to 2021. The GBD database contains 204 countries and regions, 371 diseases, and 87 risk factors. These include estimates for many different models of disease and injury outcomes. In this study, we used the GBD results tool to retrieve data on the burden of IDII attributable to Pb exposure in China. Our data are available at the national level. In this study, we filtered the GBD estimate as ‘risk factor,’ risk as ‘lead exposure,’ cause as ‘mental disorders’, location as ‘China,’ and set other options to select all. DALYs were estimated as the sum of (years lived with disability) YLDs and (years of life lost) YLLs to premature mortality. As IDII is a non-fatal disease, the GBD 2021 database does not provide information on mortality, the number of YLLs for premature death is 0, i.e., DALYs = YLDs (17). Numerical values and their 95% uncertainty intervals (UIs) were used to describe the disease burden. We summarized the DALYs trends attributed to Pb exposure in IDII in China based on age, sex, and year assessment from 1990 to 2021. Since data for GBD are anonymized and publicly available, ethical approval is not required for this study. Age-specific DALYs patterns were analyzed in 20 stratified age groups: <5, 5–9, 10–14, 15–19, 20–24, 25–29, 30–34, 35–39, 40–44, 45–49, 50–54, 55–59, 60–64, 65–69, 70–74, 75–79, 80–84, 85–89, 90–94 and 95+. The general analytical approach used in the GBD 2021 is detailed on the official GBD portal^2^.

### 2.2 Statistics

This study analyzed changes in IDII burden due to Pb exposure in China from 1990 to 2021. We used the joinpoint regression model to calculate the estimated annual percentage change (EAPC) of the global ASDR to reflect disease burden trends. The EAPC is a widely used metric in epidemiological studies, particularly for assessing temporal trends in disease burden, and it provides an intuitive quantification of both the magnitude and direction of trends (18). Compared to simple linear regression, the EAPC more effectively handle non-linear patterns, especially when disease burden exhibits variable rates of change over time. The EAPC is calculated by fitting a linear regression model to the natural logarithm of age-standardized rates (ASR), In(ASR) = *α* + *β*x + *ε*, EAPC (with 95% confidence intervals(CI)) = 100×(e^β^-1), where *x* represents calendar year, *β* represents the annual rate of change (19). An EAPC with a lower 95% confidence limit > 0 indicates a statistically significant increasing trend, whereas an upper 95% confidence limit < 0 reflects a significant decreasing trend. Otherwise, the temporal trend is considered non-significant (20, 21).

To evaluate temporal trends in age-standardized death rates (ASDR) of IDII attributable to Pb exposure during 1990–2021 in China, we applied joinpoint regression analysis to compute both the annual percentage change (APC) and average annual percentage change (AAPC), along with their corresponding 95% CI. The APC was used to identify distinct linear trend segments, whereas the AAPC provided an integrated estimate of the overall average trend spanning multiple time periods (22). Compared to EAPC, the AAPC more accurately characterizes segmented trends, thereby yielding a more robust measure of the composite rate of change. The joinpoint regression model was specified as: ln(ASDR) = *α* + βᵢx + *ε*. APC = 100 × [exp(βᵢ) - 1], AAPC = [∑(wᵢ × APCᵢ)]/(∑wᵢ); where *x* represents calendar year, *β*ᵢ represents the slope coefficient for each identified segment, *w*ᵢ is the segment-specific time span weight, A statistically significant increasing trend in ASDR was concluded when the lower bound of the AAPC’s a lower 95% confidence limit > 0. Conversely, a significant decreasing trend was identified when the upper 95% confidence limit < 0. Otherwise, the ASDR was considered stable (23).

The Age-Period-Cohort (APC) model is used to evaluate the influence of age, period, and birth cohort on outcomes, thereby describing and analyzing the incidence of diseases or mortality risks. We examined the effect of Pb exposure on the DALYs trend of IDII using the NIH APC Web Tool^3^, analyzed the Wald test using the endogenous estimator algorithm and parametric hypothesis testing. Age effects represent the biological association between aging and Pb-induced IDII development. Period effects capture temporal variations in Pb-associated IDII risk across calendar years. Cohort effects reflect how societal changes across generations modify susceptibility to Pb-related IDII. The following output parameters are derived from the APC model: (1) Net drift: the overall trend in age-standardized disease rates over time. (2) Local drifts: the trend in disease rates over time across different age groups. (3) The longitudinal age curve: disease rate changes across different ages within the same birth cohort (reflecting life-course trajectories); (4) Cross-sectional age curve: disease rate distribution across age groups during the same time period (reflecting current age-specific patterns); (5) Period rate ratio (RR): the RR for a specific period relative to the reference period. (6) Cohort RR: the cohort RR relative to the reference cohort.

Finally, we used the Bayesian age-period-cohort (BAPC) Model integrated nested Laplace approximations to predict the number and rate of disease burden attributable to IDII attributable to Pb exposure from 2022 to 2035. Compared to conventional analytical approaches, the BAPC model operates within a probabilistic inference framework, generating prediction intervals (rather than single-point estimates) for more reliable long-term (>10-year) disease burden projections (24), The BAPC framework synergistically integrates sample data with prior distributions to derive posterior estimates, maintaining robust predictive performance even with limited observational data (25). Our study employed integrated nested Laplace approximations (INLA) to circumvent the mixing and convergence challenges inherent in traditional Markov chain Monte Carlo methods. This approach significantly enhances both computational efficiency and predictive reliability, establishing a comprehensive analytical framework for burden forecasting (26). The analytical framework was implemented using the BAPC and INLA^4^ packages within the R statistical computing environment (27, 28). For parameter specification, age, period, and cohort effects were modeled using either second-order random walk processes or fixed effects, as appropriate (29). The R script for BAPC is provided in the Supplementary material 1.

All statistical analyses and plots were performed using R software (version 4.4.0). A *p*-value < 0.05 (two-sided) is the significance criterion.

## 3 Results

### 3.1 Temporal and gender trends in disease burden attributable to Pb-exposed IDII

**Figure 1:**
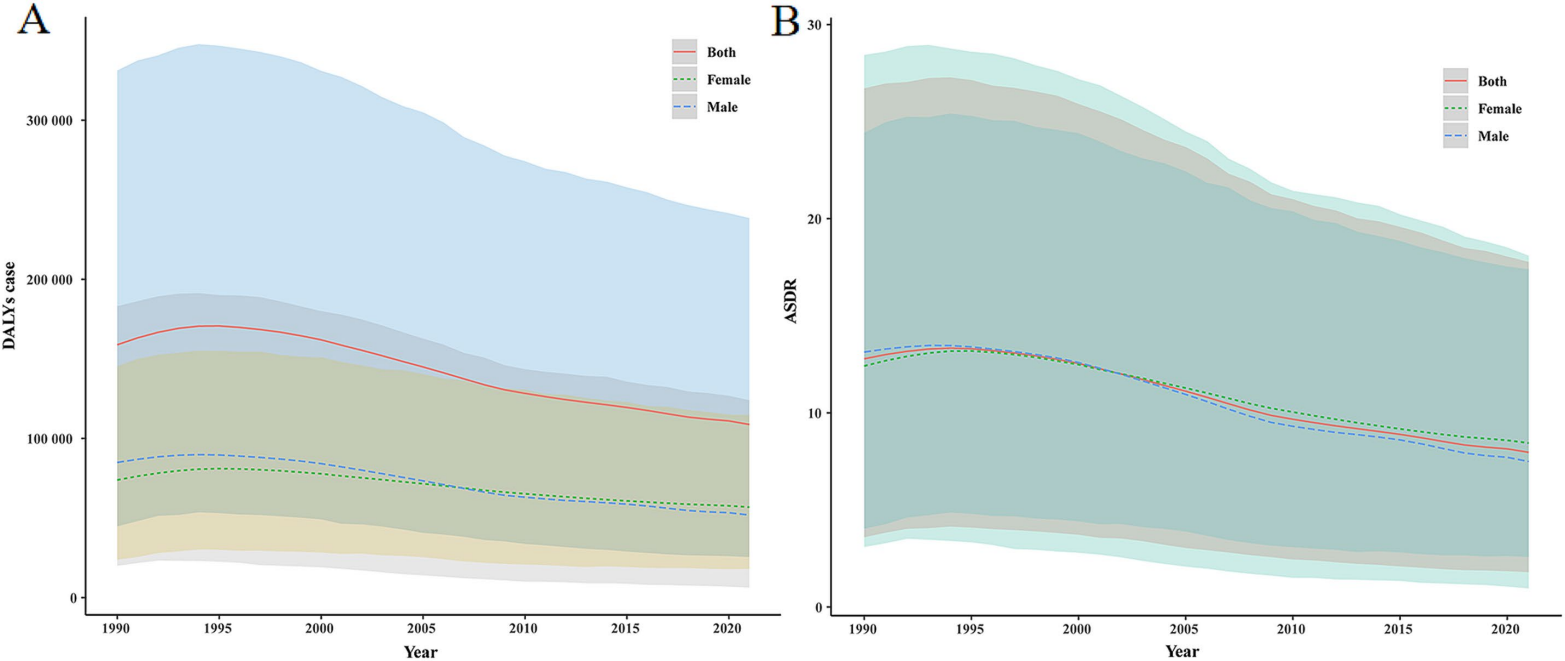
The DALYs number and ASDR of IDII attributable to Pb exposure in China from 1990 to 2021. **(A)** The DALYs number; **(B)** ASDR.

This study systematically analyzed the trends in disease burden attributable to Pb exposure-induced IDII in China from 1990 to 2021 (Table 1 and Figures 1A,B). In 1990, the number of DALYs was 158,855.019 (95%UI: 44,770.104–331,149.578), and ASDR was 12.787 per 100,000 population (95%UI: 3.596 per 100,000–26.712 per 100,000), and by 2021, the number of DALYs decreased to 108,743.995 (95%UI: 25,511.317–238,465.268), and ASDR was 7.969 per 100,000 population (95%UI: 1.798 per 100,000–17.775 per 100,000). The EAPC was −1.856% (95%CI: −2.003%–−1.710%). It demonstrated a significant and sustained downward trend in the disease burden of Pb exposure-associated IDII.

**Table 1:** The burden of idiopathic developmental intellectual disability due to Pb exposure in China from 1990 to 2021.

Index	DALYs number		ASDR per 100,000		
	1990 No. (95% UI)	2021 No. (95% UI)	1990 No. (95% UI)	2021 No. (95% UI)	EAPC No. (95% CI)
Both	158855.019 (44770.104–331149.578)	108743.995 (25511.317–238465.268)	12.787 (3.596–26.712)	7.969 (1.798–17.775)	−1.856 (−2.003–−1.710)
Sex					
Male	84932.541 (19955.458–183062.895)	51881.806 (6250.965–124037.913)	13.132 (3.097–28.434)	7.496 (0.959–18.091)	−2.115 (−2.268–−1.963)
Female	73922.477 (23831.721–145363.724)	56862.189 (18064.315–114874.227)	12.416 (4.037–24.431)	8.449 (2.580–17.388)	−1.597 (−1.742–−1.452)

DALYs, disability-adjusted life-years; No., number; UI, uncertainty interval; EAPC, estimated annual percentage change; CI, confidential interval; ASDR: age-standardized DALYs rate.

Sex-stratified analysis demonstrated statistically significant differences between males and females: for males, the decline in disease burden was more substantial (EAPC = −2.115, 95%CI: −2.268%–−1.963%), the number of DALYs was 849,32.541 (95% UI: 19,955.458–183,062.895), and ASDR was 13.132 per 100,000 population (95% UI: 3.097 per 100,000–28.434 per 100,000) in 1990. In 2021, the number of DALYs decreased to 51,881.806 (95%UI: 6,250.965–124,037.913) with an ASDR of 7.496 per 100,000 population (95%UI: 0.959 per 100,000–18.091 per 100,000). Females are also on a downward trend (EAPC = −1.597, 95%CI: −1.742%–−1.452%); the number of DALYs reached 73,922.477 (95%UI: 23,831.721–145,363.724) in 1990, with ASDR was 12.416 per 100,000 population (95%UI: 4.037 per 100,000–24.431 per 100,000); In 2021, the number of DALYs was 56,862.189 (95%UI: 18,064.315–114,874.227), with ASDR of 8.449 per 100,000 population (95%UI: 2.580 per 100,000–17.388 per 100,000). Notably, although absolute case numbers decreased substantially, the 2021 prevalence rate among females (8.449 per 100,000 population) significantly surpassed that of males (7.496 per 100,000 population). This reversal of the traditional gender disparity underscores the necessity to investigate sex-specific susceptibility to Pb exposure.

### 3.2 Age distribution characteristics of IDII disease burden attributable to Pb exposure in China, 1990–2021

**Figure 2:**
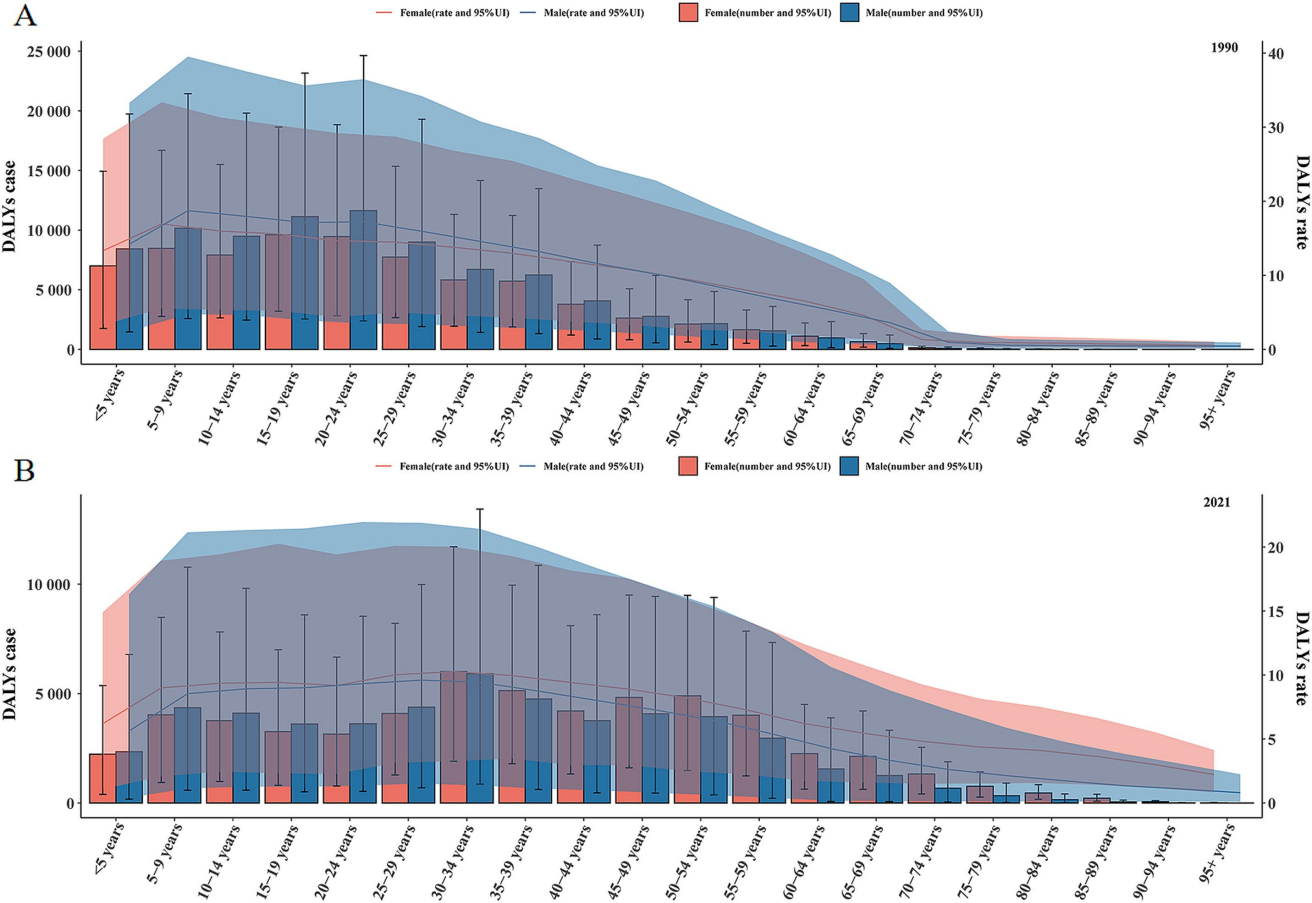
The DALYs number and rate of IDII attributable to Pb exposure in China. **(A)** The DALYs number and rate in 1990. **(B)** The DALYs number and rate in 2021.

Overall, the trend of DALYs in males and females with IDII attributable to Pb exposure in China was about the same, with an increase first and then a decline with age. In 1990, the 20–24 years age group reached a peak [21094.755 (95% UI: 5534.049–43575.754)], followed by 15–19 years [20743.520 (95% UI: 5799.155–42051.116)] and 5–9 years [18639.396 (95% UI: 5310.384–38068.429)], 25 years and above DALYs number decreases with age and plateaus over 70 years old. The DALYs rate peaks at the age of 5–9 years and then gradually declines (Table 2 and Figure 2A).

**Table 2:** The burden of idiopathic developmental intellectual disability due to Pb exposure in China from 1990 to 2021 (age group).

Age group	DALYs number		DALYs rate per 100,000		
	1990 No. (95% UI)	2021 No. (95% UI)	1990 No. (95% UI)	2021 No. (95% UI)	EAPC No. (95% CI)
<5 years	15427.820(3254.046–34621.875)	4579.032 (608.891–12118.296)	13.799 (2.910–30.966)	5.896 (0.784–15.603)	−3.185 (−3.366–−3.004)
5–9 years	18639.396 (5310.384–38068.429)	8387.277 (1902.161–19492.997)	17.875 (5.093–36.507)	8.758 (1.986–20.354)	−2.639 (−2.803–−2.474)
10–14 years	17389.286 (5048.766–35163.418)	7868.103 (1653.212–17644.113)	17.000 (4.936–34.375)	9.128 (1.918–20.470)	−2.219 (−2.376–−2.061)
15–19 years	20743.520 (5799.155–42051.116)	6872.836 (1357.861–15548.711)	16.377 (4.578–33.199)	9.204 (1.818–20.823)	−2.082 (−2.185–−1.978)
20–24 years	21094.755 (5534.049–43575.754)	6778.919 (1480.629–15492.766)	15.981 (4.192–33.012)	9.264 (2.023–21.172)	−2.088 (−2.217–−1.958)
25–29 years	16740.988 (4799.752–34191.339)	8477.325 (2000.693–18124.445)	15.234 (4.368–31.114)	9.802 (2.313–20.957)	−1.854 (−2.016–−1.691)
30–34 years	12529.904 (3363.841–25439.594)	11920.421 (3340.981–25549.952)	14.199 (3.812–28.829)	9.839 (2.758–21.089)	−1.619 (−1.825–−1.414)
35–39 years	11961.204 (3280.116–24778.364)	9893.075 (2618.778–20798.287)	13.095 (3.591–27.128)	9.336 (2.471–19.628)	−1.395 (−1.571–−1.218)
40–44 years	7859.937 (2093.317–16201.423)	7963.621 (2078.810–16894.613)	11.715 (3.120–24.147)	8.700 (2.271–18.457)	−1.171 (−1.316–−1.027)
45–49 years	5402.159 (1416.458–11309.842)	8904.562 (2227.609–18698.802)	10.465 (2.744–21.910)	8.071 (2.019–16.949)	−1.114 (−1.243–−0.984)
50–54 years	4284.063 (1025.867–9017.961)	8848.865 (1948.588–18783.422)	8.979 (2.150–18.901)	7.322 (1.612–15.542)	−1.042 (−1.192–−0.891)
55–59 years	3212.712 (784.414–6918.080)	6971.893 (1594.148–15089.906)	7.408 (1.809–15.952)	6.341 (1.450–13.725)	−0.982 (−1.171–−0.792)
60–64 years	2082.547 (500.664–4542.920)	3818.703 (724.795–8315.583)	5.893(1.417–12.856)	5.231 (0.993–11.390)	−0.831 (−1.047–−0.615)
65–69 years	1127.001 (273.698–2479.852)	3391.131 (746.649–7382.762)	4.131 (1.003–9.090)	4.421 (0.973–9.625)	−0.402 (−0.721–−0.082)
70–74 years	218.060 (59.922–475.309)	2005.086 (475.867–4409.442)	1.159 (0.318–2.526)	3.762 (0.893–8.273)	1.439 (0.324–2.567)
75–79 years	89.011 (25.743–189.555)	1101.715 (314.145–2305.511)	0.782 (0.226–1.666)	3.327 (0.949–6.961)	2.371 (1.131–3.625)
80–84 years	39.148 (12.140–78.487)	609.183 (195.615–1209.258)	0.739 (0.229–1.482)	3.078 (0.988–6.110)	5.888 (4.943–6.842)
85–89 years	11.417 (3.559–23.074)	267.252 (94.501–519.696)	0.677 (0.211–1.368)	2.806 (0.992–5.456)	6.183 (5.329–7.044)
90–94 years	1.880 (0.584–3.731)	72.485 (27.287–138.804)	0.613 (0.190–1.216)	2.472 (0.931–4.734)	4.601 (3.623–5.588)
95+ years	0.213 (0.067–0.420)	12.510 (4.582–24.152)	0.525 (0.165–1.037)	1.957 (0.717–3.779)	3.271 (2.175–4.379)

DALYs, disability-adjusted life-years; No., number; UI, uncertainty interval; EAPC, estimated annual percentage change; CI, confidential interval.

In 2021, the DALYs number of IDII attributable to Pb exposure in China showed a fluctuating trend, with the highest point in 30–34 years [11,920.421 (95% UI: 3,340.981–25,549.952)], followed by 35–39 years [9,893.075 (95% UI: 2,618.778–20,798.287)] and 45–49 years [8,904.562 (95% UI: 2,227.609–18,698.802)]. The DALYs rate peaks at the age of 30–34 years [95%UI: 9.839/100,000 (2.758/100,000–21.089/100,000)] and then slowly declines. Unlike in 1990, females consistently had a higher DALYs rate than males in 2021 (Table 2 and Figure 2B). Compared with 1990, a significant rightward shift in the age spectrum of disease burden attributable to Pb exposure-induced IDII.

### 3.3 AAPC analysis of DALYs of IDII attributable to Pb exposure

**Figure 3:**
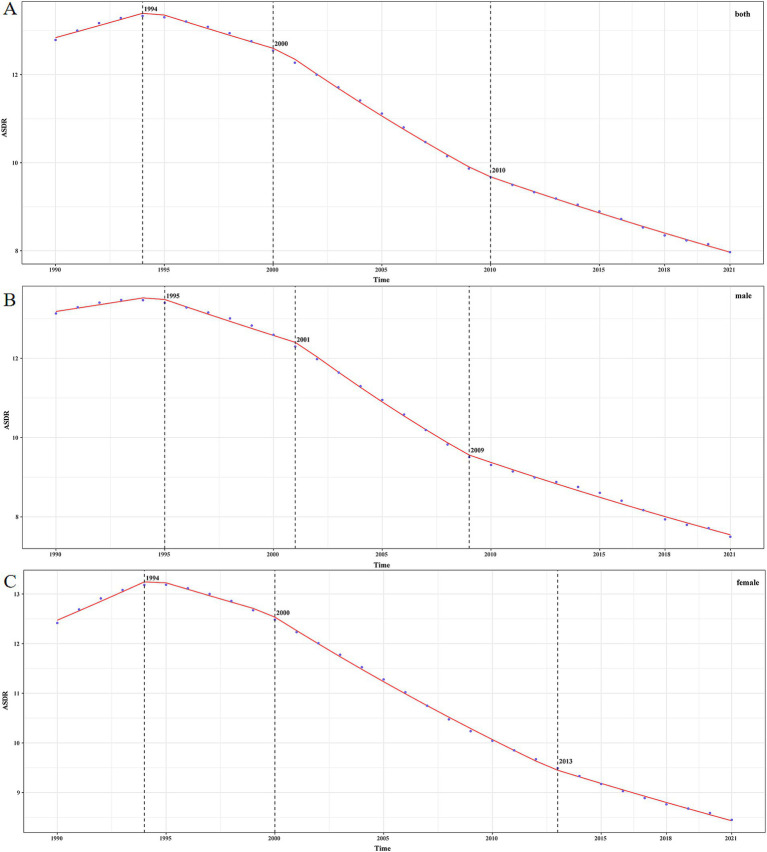
The ASDR of IDII attributable to Pb exposure in China from 1990 to 2021. **(A)** Both sexes; **(B)** male; **(C)** female.

As shown in Figure 3A and Table 3, the overall trend of the burden of IDII attributed to Pb exposure is downward [AAPC_1990–2021_ = −0.157 (95% CI: −0.159, −0.155)]. ASDR of both male and female significantly increased at first, then considerably declined after 1994. In males and females, the burden of IDII attributable to Pb exposure rises and then decreases. In males (Figure 3B and Table 3), ASDR underwent one significant increase and three significant declines. In females (Figure 3C and Table 3), ASDR underwent one significant increase, and three significant declines Over the entire study period, AAPC was [AAPC_1990–2021_ = −0.182 (95% CI: −0.185, −0.179)] in males and [AAPC_1990–2021_ = −0.130 (95% CI: −0.133, −0.128)] in females. It can be seen that both males and females were in the period 2000–2010 when the APPC of ASDR declined rapidly.

**Table 3:** The APC of idiopathic developmental intellectual disability due to Pb exposure in China from 1990 to 2021.

Index	Period	APC% (95% CI)	*P*	AAPC% (95%CI)
Both (1/100000)	1990–1994	1.060 (0.820, 1.300)	<0.05	−0.157 (−0.159, −0.155)
	1994–2000	−1.157 (−1.335, −0.980)	<0.05	
	2000–2010	−2.720 (−2.815, −2.626)	<0.05	
	2010–2021	−1.753 (−1.814, −1.691)	<0.05	
Male (1/100000)	1990–1995	0.643 (0.240, 1.048)	<0.05	−0.182 (−0.185, −0.179)
	1995–2001	−1.383 (−1.619, −1.147)	<0.05	
	2001–2009	−3.254 (−3.485, −3.021)	<0.05	
	2009–2021	−1.951 (−2.043, −1.859)	<0.05	
Female (1/100000)	1990–1994	1.512 (1.280, 1.745)	<0.05	−0.130 (−0.133, −0.128)
	1994–2000	0.987 (−1.214, −0.760)	<0.05	
	2000–2013	2.169 (−2.222, −2.116)	<0.05	
	2013–2021	−1.415 (−1.507, −1.322)	<0.05	

APC, average percentage change; APPC, annual average percentage change.

### 3.4 APC analysis of ASDR of IDII attributable to Pb exposure

**Figure 4:**
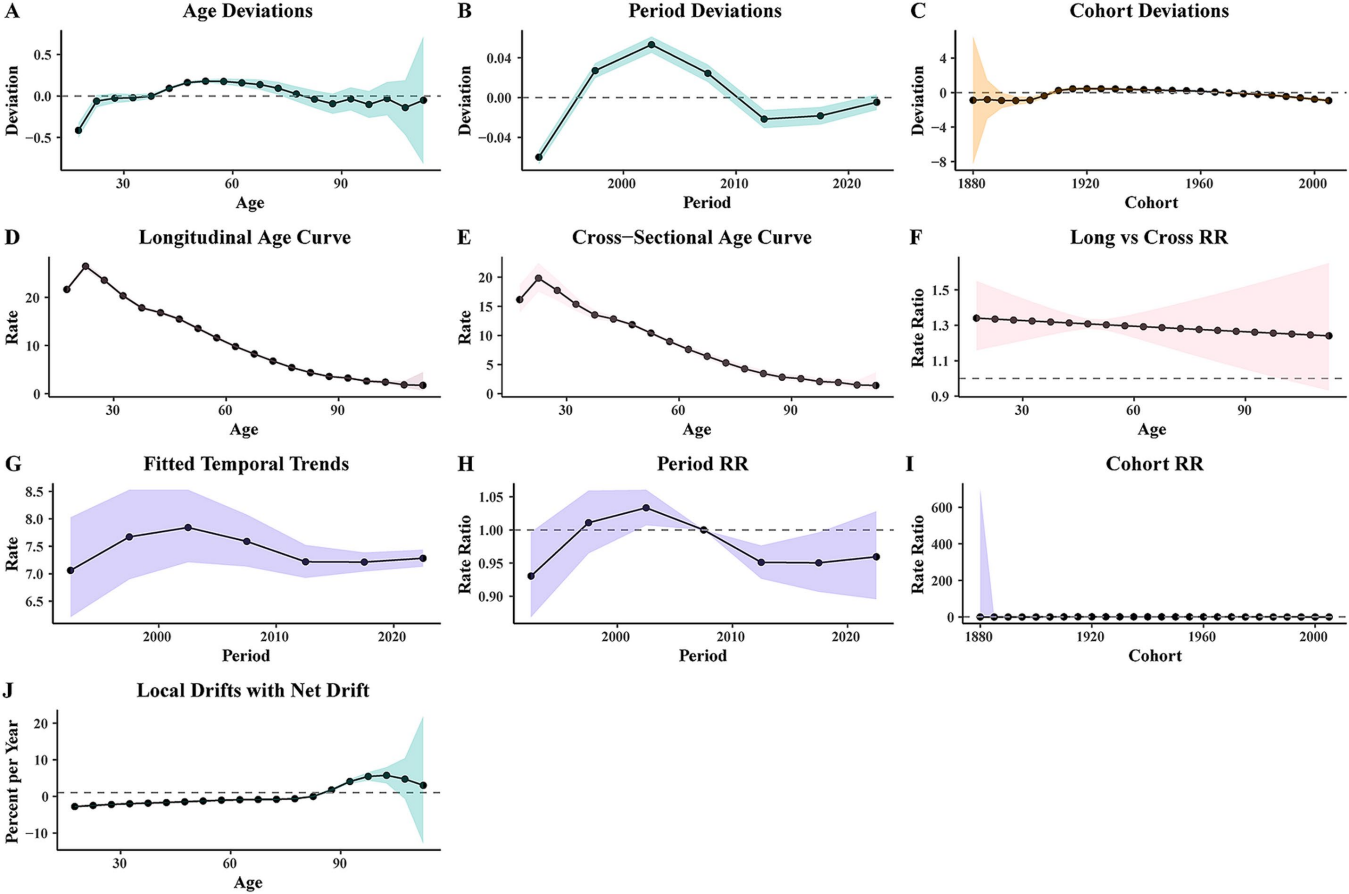
The ASDR of APC model of IDII attributable to Pb exposure in China. **(A)** Age deviation; **(B)** period deviation; **(C)** cohort deviation; **(D)** longitudinal age curve; **(E)** cross-sectional age curve; **(F)** long vs. cross RR; **(G)** fitted temporal trends; **(H)** period RR; **(I)** cohort RR; **(J)** Local drifts with net drift.

An APC model was used to analyze the ASDR in the Chinese population from 1990 to 2021, with the model fitting results illustrated in Figure 4. The age deviation (Figure 4A) results showed that with the increase of age, the deviation value of the incidence of diseases in Chinese residents from 1990 to 2021 increased first and then decreased, and finally fluctuated, with the highest point increasing by 2.324 times compared with the lowest point. The period deviation model (Figure 4B) demonstrated a N-shaped non-linear temporal trend in IDII DALYs attributable to Pb exposure in China from 1990 to 2021, and the highest points 1.1273 times higher than the lowest point. The cohort deviation model showed (Figure 4C) that the risk of disease increased as the birth cohort progressed and then stabilized. With age, the values of both the longitudinal age curve and the cross-sectional age curve first increased, reaching a maximum at 22.5 years of age and then gradually decreasing (Figures 4D,E). The value of the long vs. cross rate ratios (RR) demonstrated an age-dependent decreasing trend (Figure 4F). The results of the fitted temporal trends model (Figure 4G) showed that the incidence of the disease also increased and then decreased over time, reaching 7.843. In the Period RR model (Figure 4H), the value also rose first in 2002, then reduced, and then showed an upward trend. In the cohort RR model (Figure 4I), it grew steadily, then fell steadily, with the highest point in 1925. In the Local Drifts with Net Drift model (Figure 4J), the percentage of risk increases with age. The analysis revealed three findings: (1) the disease risk peaked during adolescence (age 22.5 years); (2) a significant transition in disease burden was observed circa 2002; (3) the high risk in the 1925 birth cohort likely reflects historically Pb exposure profiles.

### 3.5 Trends of ASDR and DALYs predicted by BAPC

**Figure 5:**
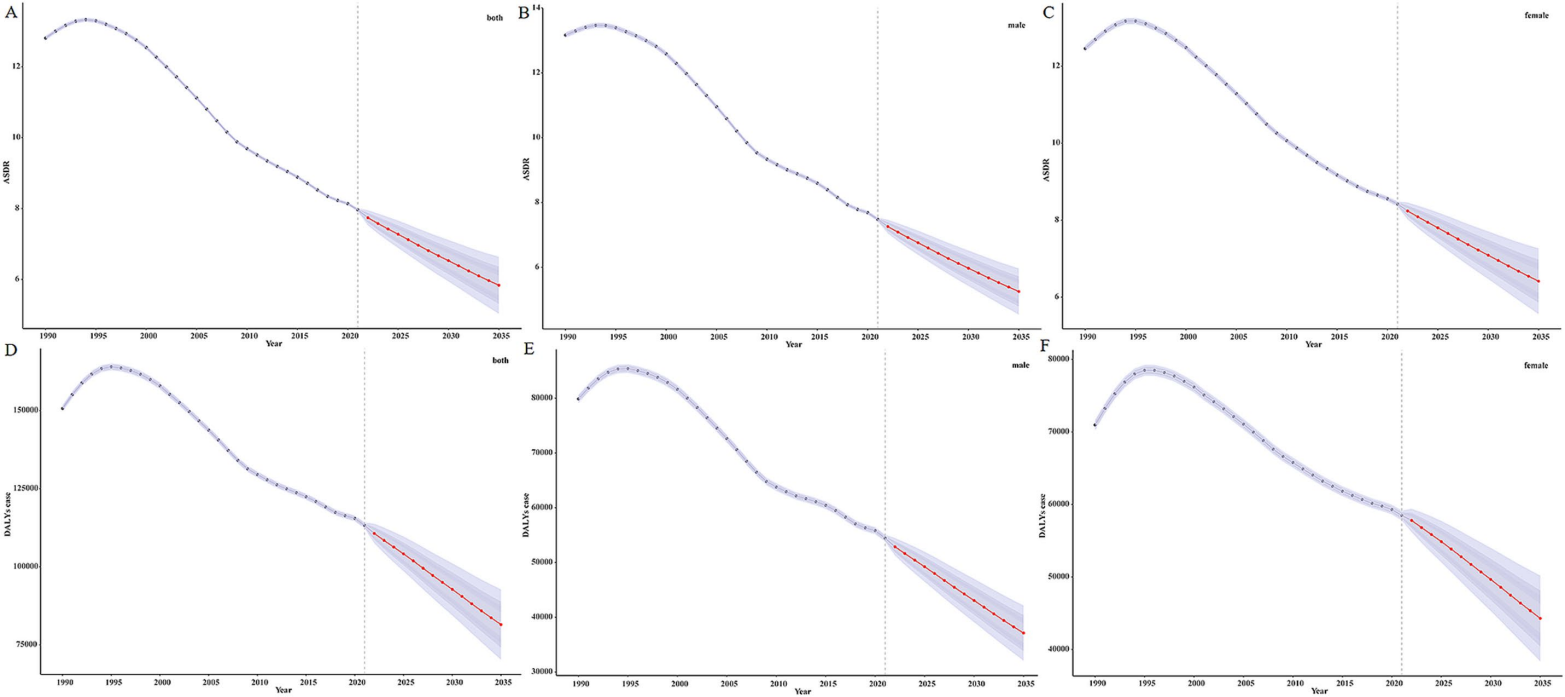
The DALYs and ASDR of BAPC model of IDII attributable to Pb exposure in China. **(A)** ASDR of both sexes; **(B)** ASDR of male; **(C)** ASDR of female; **(D)** DALYs number of both sexes; **(E)** DALYs number of male; **(F)** DALYs number of female.

We used the BAPC model to conduct sex- and age-subgroup predictive analyses of disease burden attributable to Pb exposure-induced IDII among Chinese population from 2021 to 2035 (Figure 5). The projection results demonstrate a consistent downward trend in ASDR of IDII attributable to Pb exposure in China (Figure 5A). Notably, by 2035, the predicted ASDR values are projected to reach 5.254 per 100,000 population for males and 6.414 per 100,000 population for females (Figures 5B,C). We also find that the DALYs number in the total population, and male and female in China showed a fluctuating downward trend, and it is expected to reach 81504.769, 37112.184, and 44293.649, respectively, by 2035 (Figures 5D–F).

**Figure 6:**
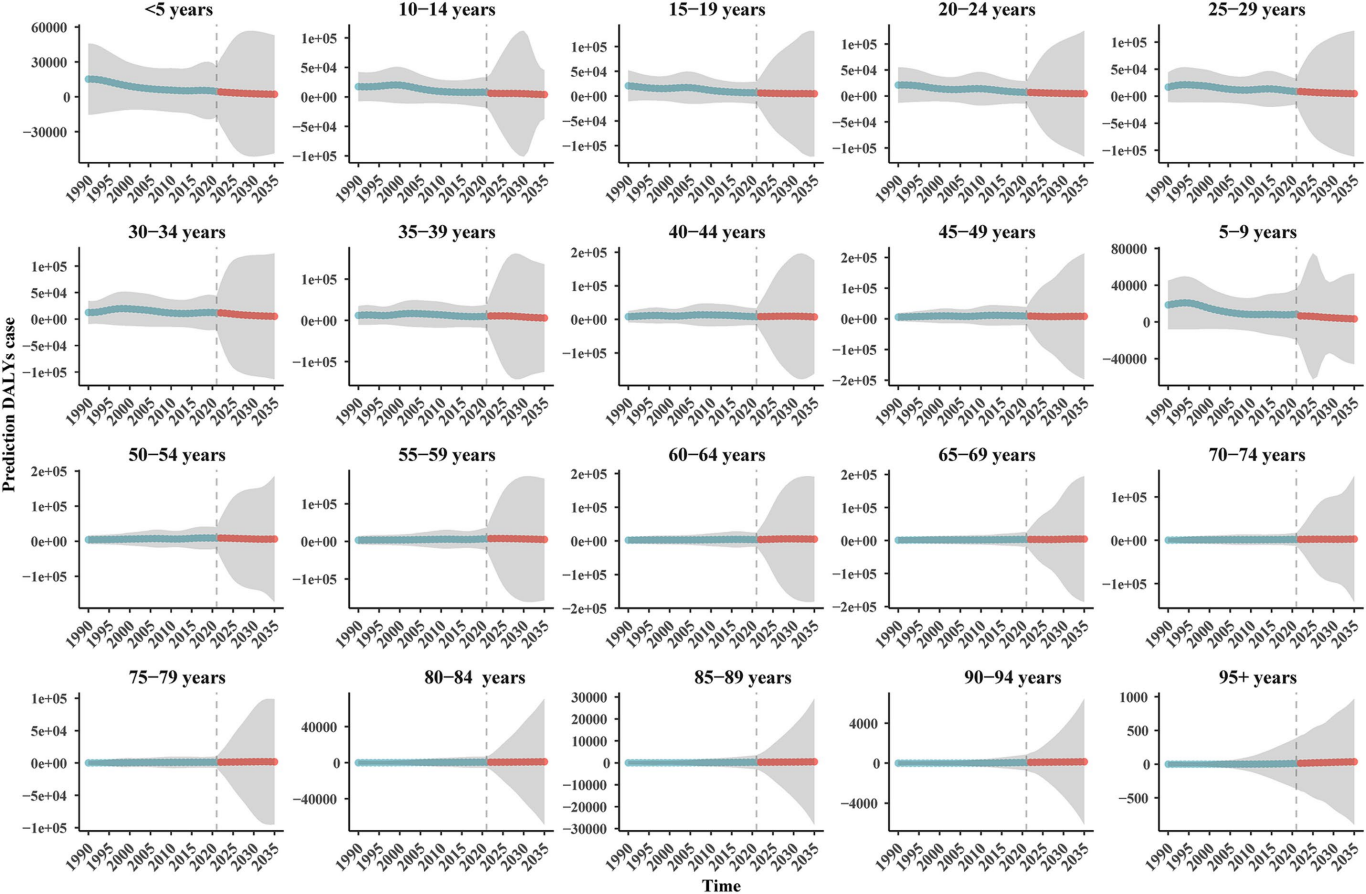
The prediction DALYs number of BAPC model of IDII attributable to Pb exposure in China.

**Figure 7:**
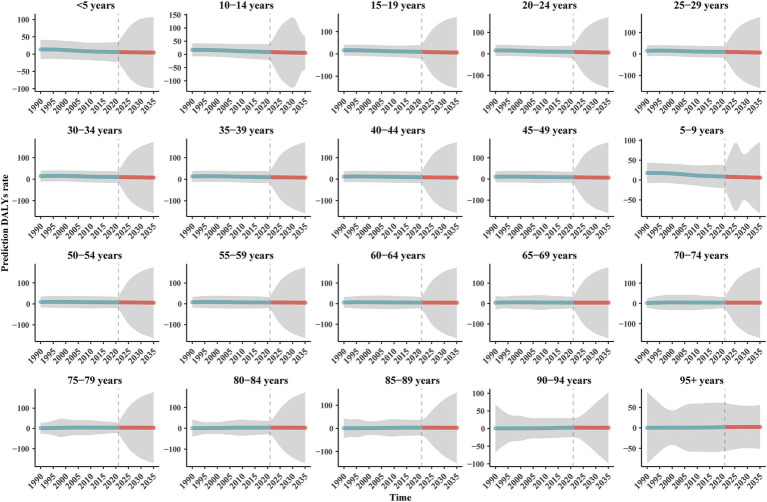
The prediction DALYs rate of BAPC model of IDII attributable to Pb exposure in China. (H) Prediction DALYs rate.

We also predicted the DALYs number and rate for different age groups (Figures 6, 7). The DALYs number in the 12 age groups less than or equal to 55–59 years showed a downward trend. In the groups of 60–64 years and 65–69 years, the DALYs number decreased first and then increased, and in 2035, the overall DALYs number was rising, compared with 2021. The number of DALYs in six age groups over 70–74 years showed an upward trend. However, the DALYs rate for each age group is on a downward trend.

### 3.6 Ranking of the contribution of Pb exposure to the burden of disease

**Figure 8:**
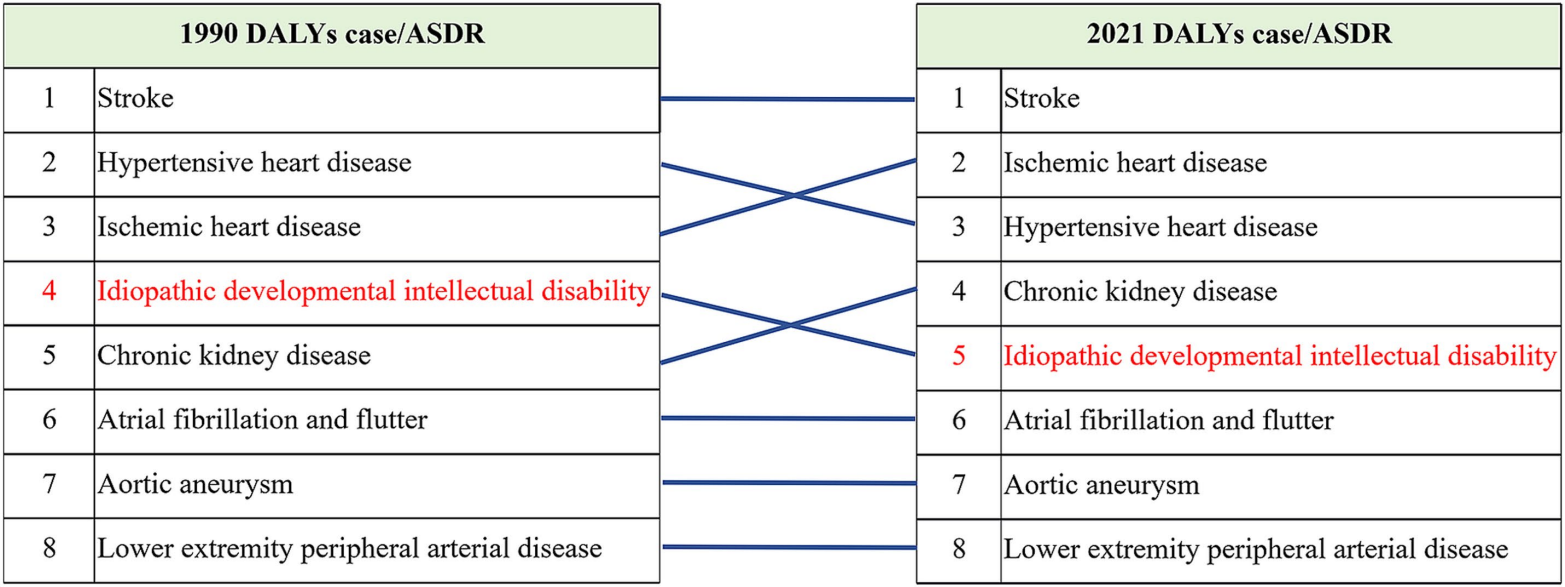
Ranking of the contribution of Pb exposure to the burden of disease.

From 1990 to 2021, the ranking of diseases caused by Pb exposure based on DALYs has changed (Figure 8): among the diseases, stroke has been firmly in the first place, with hypertensive heart disease and IDII rankings declining, while ischemic heart disease and chronic kidney disease rankings have risen, and atrial fibrillation and flutter, aortic aneurysm and lower extremity peripheral arterial disease rankings have remained unchanged.

## 4 Discussion

This study provides the first systematic evaluation of long-term epidemiological characteristics of disease burden attributable to Pb exposure-induced IDII in China. Analysis of 1990–2021 data revealed decline trends in both DALYs and ASDR for Pb exposure-related IDII. The ranking of IDII declined from fourth to fifth among Pb exposed diseases, a change that was accompanied by significant reductions in both ASDR and DALYs, with males demonstrating a markedly faster rate of decline compared to females. This change may originate from demographic and epidemiological transitions such as reduced occupational exposure, along with national environmental policy adjustments (16, 30). In developing countries, primary Pb exposure sources include environmental pollution, industrial exposure, and food contamination (31, 32). Since the phase-out of Pb gasoline, the environment for Pb exposure have shown marked improvement, with global atmospheric and human blood Pb levels have reduced dramatically (33). Socio-economic development has also contributed to the reduction of the burden of disease. Maheswari et al. (34) found that nutritional supplementation and the implementation of public health measures can lower blood Pb levels and improve cognitive function. Meanwhile, children from lower socioeconomic households are more susceptible to ID, closely linked to inadequate educational resources and medical support, which can affect children’s cognitive development (35).Compared to 1990, China had attained enhanced international standing, significant socioeconomic progress, substantial healthcare system upgrades, and improved population nutritional status by 2021 (36). Combined with increased public awareness of Pb hazards (15), these factors collectively contributed to the reduction in Pb exposure-related disease burden. Notably, rapid urbanization may influence IDII incidence by altering exposure patterns, but this hypothesis requires further epidemiological evidence for validation.

This study reveals gender-specific characteristics in the disease burden associated with Pb exposure. Longitudinal analysis demonstrates that while both male and female populations show sustained declining trends in DALYs, the rate of decline is faster in males than in females. More importantly, the gender disparity in ASDR progressively widens over time, indicating that current public health interventions have produced more prominent protective effects in the male population. These differences may be attributable to the significant role of gender in Pb toxicity and biological metabolic processes (37). The Pb that enters the human body is primarily stored in the bone, and the biological half-life of inorganic Pb in the bones is approximately 10–30 years (38). Unlike males, females exhibit slower bone Pb release rates, maintaining elevated blood Pb levels 2–3 times longer than males after cessation of external exposure, creating a persistent endogenous exposure source (37). Pb exposure can prolong reaction time, especially in females (39). Although the prevalence of hyperuricemia in Chinese adults is much higher in males than in females, the prevalence in females is higher than in males in Pb-contaminated areas. Persistent Pb exposure affects females more than males, and among participants, females are older than males (40). Animal experiments further demonstrate that Pb exposure only increased the sensitivity of female mice to delta-9-tetrahydrocannabinol but had no effect on male mice (41), and causes more severe and prolonged Pb accumulation in the female hippocampus (42). It may be because Pb exposure has a more extended incubation period for adverse effects in females, but it is higher in females than in males. These findings suggest that Pb’s neurotoxic effects on females may have longer latency periods and greater persistence.

The disease burden of Pb-induced IDII in China exhibits significant age-distribution evolution. In 1990, the disease burden analysis showed Pb-related IDII mainly concentrated in children and adolescents, possibly due to the fact that Pb crosses the placental barrier when the mother is exposed to Pb undetected during pregnancy, potentially impairing fetal neurodevelopment (43). The second is that children’s hand-to-mouth behaviors increasing exposure risks from contaminated objects (44). However, in 2021, the disease was mainly concentrated in the age groups of 30–34 years, 35–39 years, 50–54 years, etc., and by 2035, the DALYs number in the age group of only > 60 age years will be higher than that in 2021, and there was a trend of change from adolescents to 30-year-olds and middle-aged people. This epidemiological shift may stem from multiple factors: firstly, the structural change of the Chinese population has had an impact on IDII, the decline in fertility has led to a decrease in the number of newborns, but the proportion of older adult has increased, and this demographic change has affected the number and type of IDII. Secondly, Pb’s bioaccumulate properties create cohort effects, with aging populations sustaining endogenous exposure. Lastly, occupational exposure patterns differ substantially, with working-age adults facing higher environmental Pb risks due to outdoor activities.

The predictive analysis based on the BAPC model indicates that China’s disease burden of Pb-induced IDII will continue to decline from 2022 to 2035, characterized by reductions in both the number of DALYs and ASDR. According to research, from 2015 to 2060, the number of people aged 65 and over will increase significantly, while the number of people suffering from cognitive impairment or restrictions on activities of daily living will also increase significantly (45). Our study projects that by 2035, the target population characteristics will undergo transformations, manifested by females surpassing males in disease burden, persistently elevated Pb-associated IDII risks among the older adult aged ≥60 years, and the shifting of the disease burden peak from children/adolescents to middle-aged/older adult groups. This age and sex distribution transition suggests current Pb exposure prevention strategies require adjustment, particularly emphasizing exposure monitoring and interventions for occupational populations and the older adult.

Based on these findings, we recommend that public health authorities prioritize the following prevention and control strategies: (1) clinicians should strengthen blood Pb monitoring, particularly for occupational groups and older adult individuals over 60, by incorporating Pb exposure screening into routine health examinations. (2) According to the characteristics of female bone Pb release, develop nutritional intervention programs; guidelines for Pb therapy are designed for older populations, taking into account age-related factors such as declining renal function. (3) More stringent Pb exposure control policies should be implemented, including revisions to occupational exposure limits and stricter regulation of Pb levels in environmental media (air, soil, and water). (4) Enhanced assessments of high-risk sites, especially redevelopment projects in traditional industrial zones, and standardized management of emerging pollution sources like e-waste recycling are also crucial. (5) There is an urgent need for research on the health impacts of Pb exposure in middle-aged and older adult. (6) Public health education and risk communication should be improved to raise awareness of Pb exposure hazards, particularly among high-risk groups, and to promote dietary approaches that facilitate Pb excretion, thereby reducing its harmful effects.

There are some limitations in this study because the data was derived from the GBD database, and the estimates are derived from different quality data. However, data processing and modeling have improved, and the accuracy of forecasts has improved. In addition, these data are still definitely different from the actual data. Still, with the continuous update of the GBD database and the constant improvement of related methods, the estimates based on GBD will be more accurate and stable.

## 5 Conclusion

In summary, this investigation provides the most comprehensive evaluation of China’s Pb attributable IDII burden. Our results document a significant decline in Pb-associated IDII disease burden (both DALYs and ASDR) during 1990–2021, with projections indicating continued reduction through 2035. Notably, the burden distribution is undergoing substantial transformation, females are emerging as the predominantly affected demographic, superseding males, while the peak burden transitions from adolescent to middle-aged populations. Based on these observations, we propose public health authorities should emphasize: (1) enhanced biological monitoring targeting middle-aged occupational groups, particularly women in high-risk industries; (2) development of optimized screening protocols that account for both workplace and environmental exposure pathways; and (3) formulation of stratified prevention strategies incorporating gender- and age-subgroup considerations. Implementation of these evidence-based measures will facilitate continued reduction of Pb-related IDII burden and advance population health outcomes in China.

## Data Availability

The datasets presented in this study can be found in online repositories. The names of the repository/repositories and accession number(s) can be found below: All the data may be available from the IHME website (https://vizhub.healthdata.org/gbd-results/).

## Footnotes

^1^ https://vizhub.healthdata.org/gbd-results/

^2^ https://ghdx.healthdata.org/gbd-2021

^3^ https://analysistools.cancer.gov/apc/

^4^ https://www.r-inla.org/
