# Supplementary material for: Burdens of idiopathic developmental intellectual disability attributable to lead exposure from 1990 to 2021 and projection to 2035 in China: findings from the 2021 global burden of disease study
Source: Front Public Health. 2025 May 9;13:1562794. doi: 10.3389/fpubh.2025.1562794 (PMC12098424; doi:10.3389/fpubh.2025.1562794)
Supplement: Supplementary file 1 [file Data_Sheet_1.pdf]

Burdens of Idiopathic developmental intellectual disability attributable to Lead exposure from 1990 to 2021 and projection to 2035 in China: Findings from the 2021 Global Burden of Disease Study

Supplementary Material 1: R script used for the statistical analyses for BAPC model

```
# load the library BAPC
library(BAPC)

# read the mortality and population counts.
# The rownames (i.e. periods 1950-2007) are given in the first column.
counts = read.table("us_data_2014.txt", row.names=1, header=F)
pop = read.table("us_pop_2014.txt", row.names=1, header=F)

# define the labels for the 20 age groups
agegroup = c(" < 5", "5-9", "10-14", "15-19", "20-24", "25-29", "30-34", "35-39", "40-44", "45-49",
"50-54", "55-59", "60-64", "65-69", "70-74", "75-79", "80-84" "85-89", "90-94, "95 + ")

# define an object of class APCList were you specify
# the dataset together with the grid factor (here M=5)
# and the labels of the age groups.
us.APC = APCList(counts, pop, gf=5, agelab=agegroup)

# generate Figure 1
col <- c("grey20", "grey35", "grey50", "grey65", "grey75", "grey85")
ratesByAge(us.APC, scale=100000, age=seq(27,82,5), per=1950:2007, col=col)

# perform retrospective projection for 15 years, see section 5
us.res = BAPC(us.APC, predict=list(npredict=15, retro=TRUE))

# to generate figure 2 in the paper use the following command
plotBAPC(us.res, scale=100000, type="ageSpecBoth",
    coladd="grey80", showdata=TRUE)
```
